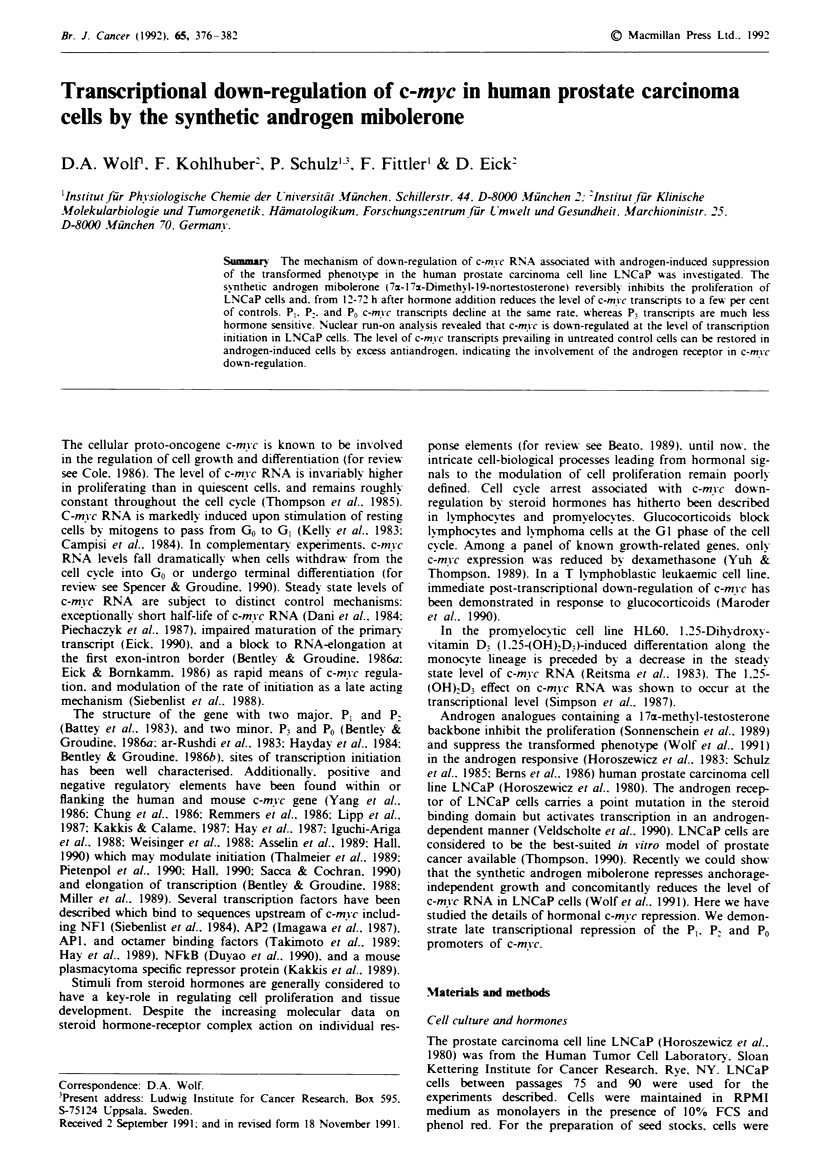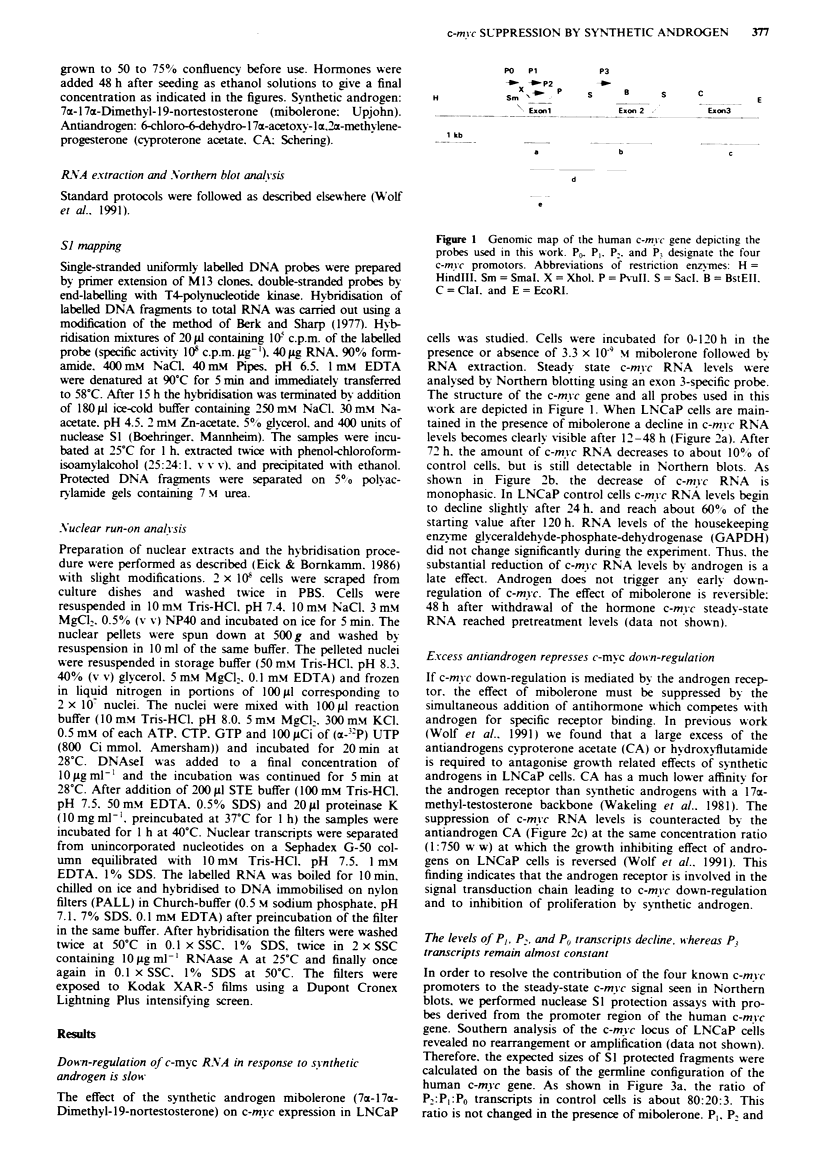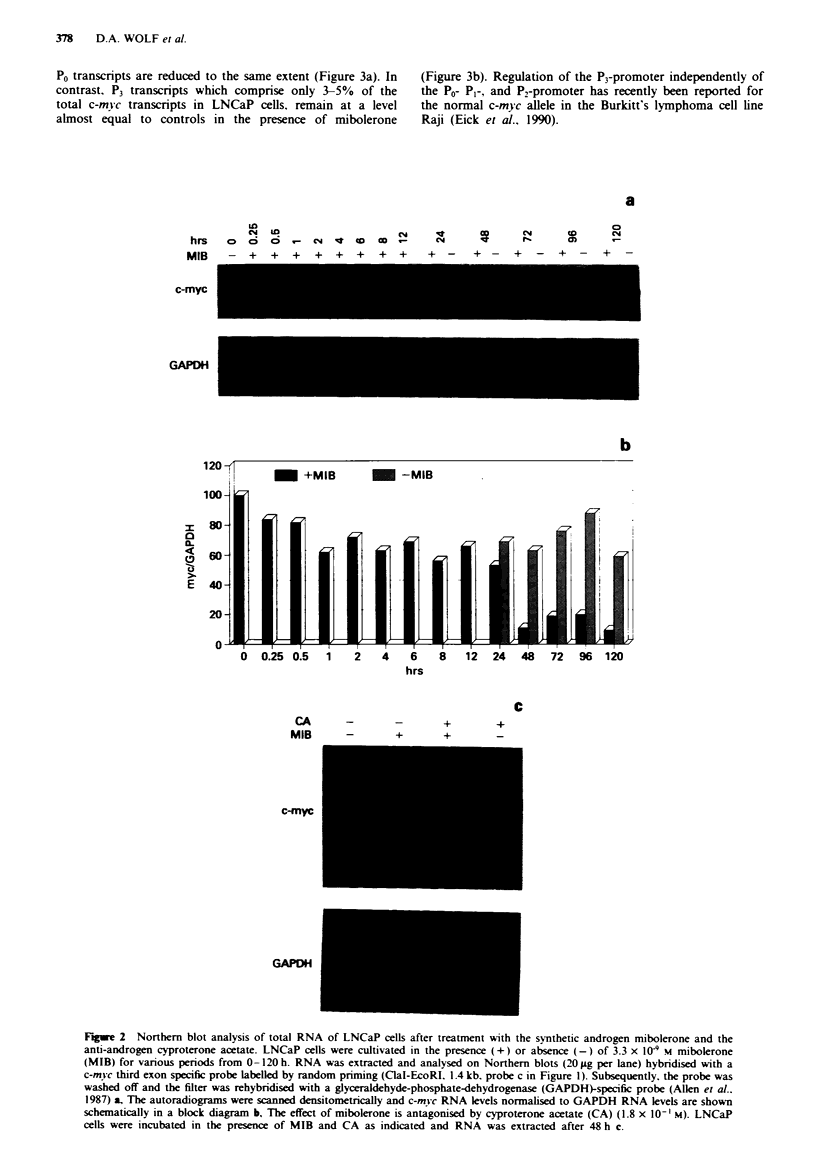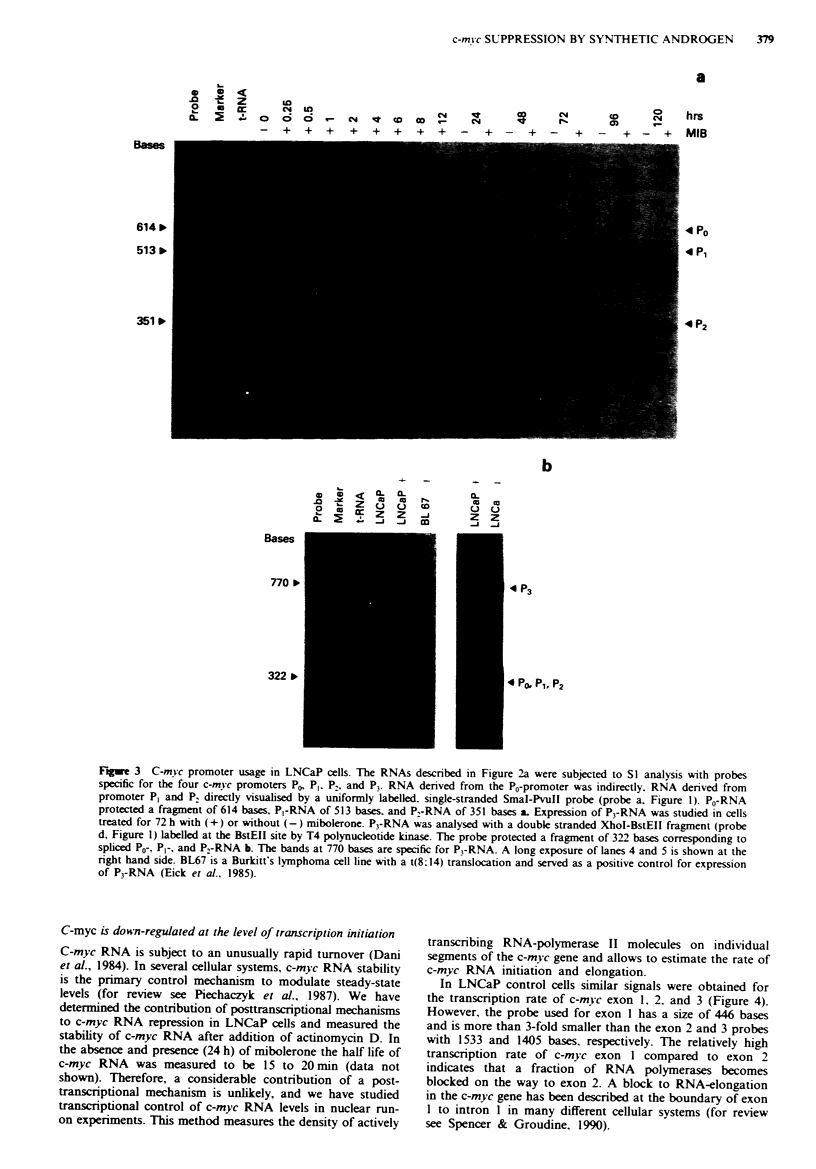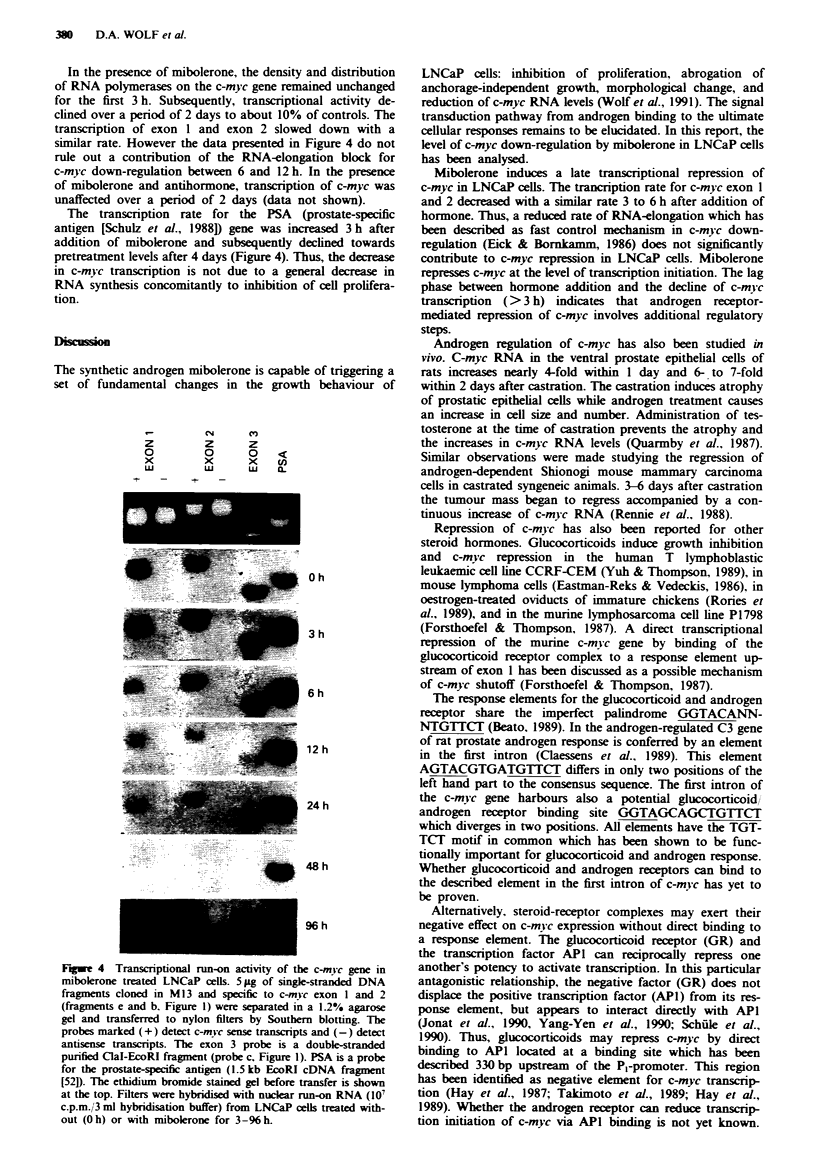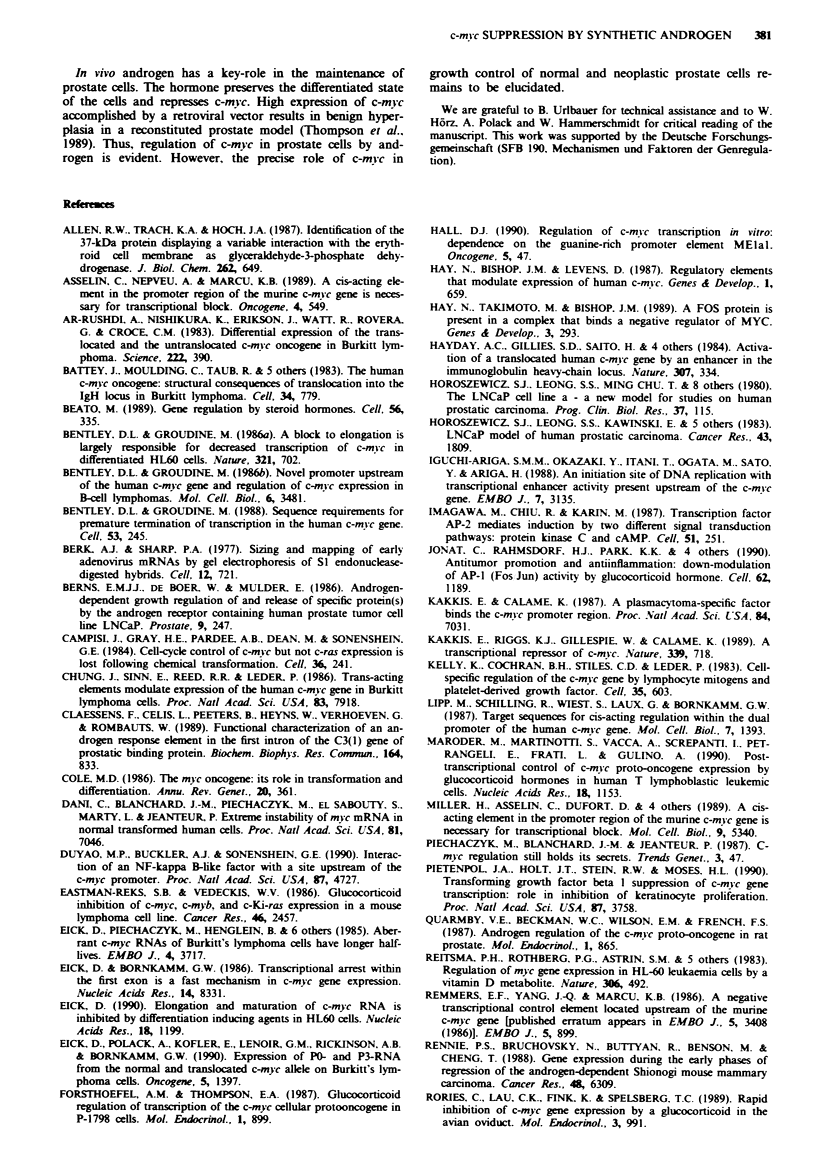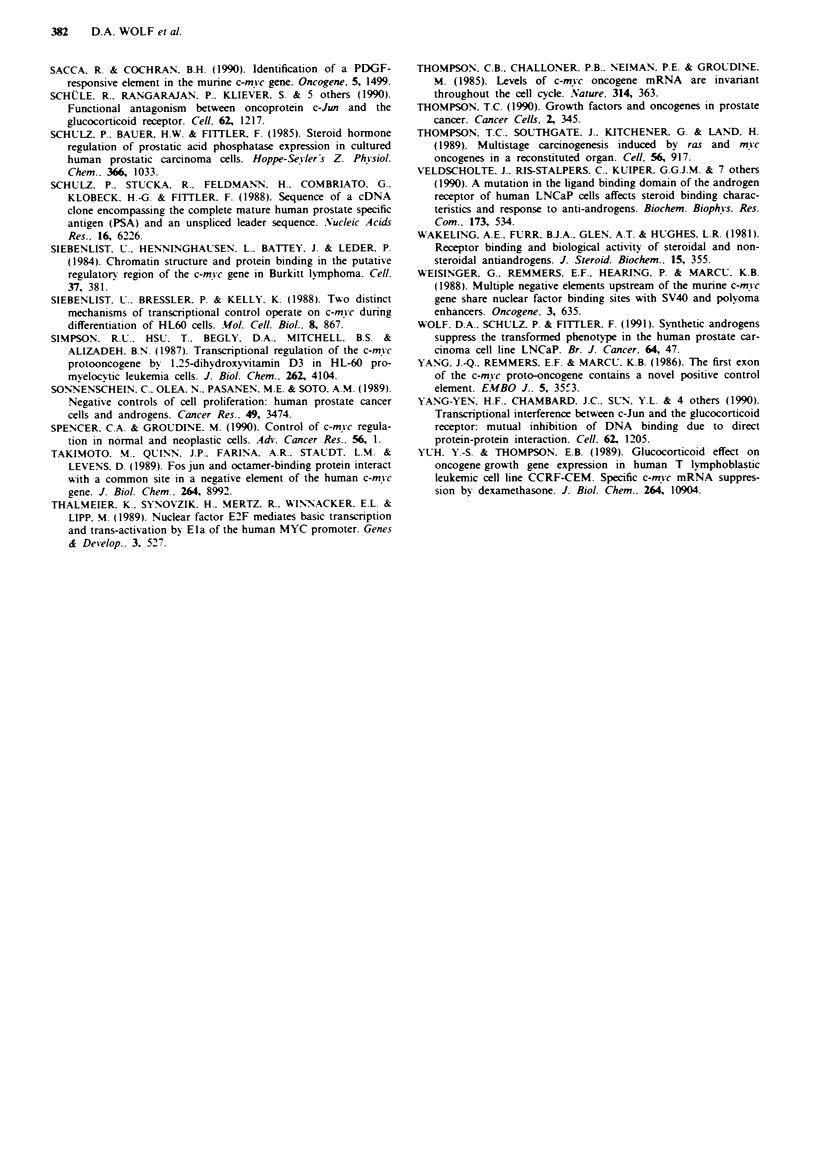# Transcriptional down-regulation of c-myc in human prostate carcinoma cells by the synthetic androgen mibolerone.

**DOI:** 10.1038/bjc.1992.76

**Published:** 1992-03

**Authors:** D. A. Wolf, F. Kohlhuber, P. Schulz, F. Fittler, D. Eick

**Affiliations:** Institut für Physiologische Chemie, Universität München, Germany.

## Abstract

**Images:**


Br. J. Cancer (1992), 65, 376-382 © Macmillan Press Ltd., 1992

### Transcriptional down-regulation of c-*myc* in human prostate carcinoma cells by the synthetic androgen mibolerone

D.A. Wolf[1], F. Kohlhuber[2], P. Schulz[1,3], F. Fittler[1] & D. Eick[2]

[1]*Institut für Physiologische Chemie der Universität München, Schillerstr. 44, D-8000 München 2;* [2]*Institut für Klinische Molekularbiologie und Tumorgenetik, Hämatologikum, Forschungszentrum für Umwelt und Gesundheit, Marchioninistr. 25, D-8000 München 70, Germany.*

**Summary** The mechanism of down-regulation of c-*myc* RNA associated with androgen-induced suppression of the transformed phenotype in the human prostate carcinoma cell line LNCaP was investigated. The synthetic androgen mibolerone (7α-17α-Dimethyl-19-nortestosterone) reversibly inhibits the proliferation of LNCaP cells and, from 12-72 h after hormone addition reduces the level of c-*myc* transcripts to a few per cent of controls. $P_1$, $P_2$, and $P_0$ c-*myc* transcripts decline at the same rate, whereas $P_3$ transcripts are much less hormone sensitive. Nuclear run-on analysis revealed that c-*myc* is down-regulated at the level of transcription initiation in LNCaP cells. The level of c-*myc* transcripts prevailing in untreated control cells can be restored in androgen-induced cells by excess antiandrogen, indicating the involvement of the androgen receptor in c-*myc* down-regulation.

The cellular proto-oncogene c-*myc* is known to be involved in the regulation of cell growth and differentiation (for review see Cole, 1986). The level of c-*myc* RNA is invariably higher in proliferating than in quiescent cells, and remains roughly constant throughout the cell cycle (Thompson *et al.*, 1985). C-*myc* RNA is markedly induced upon stimulation of resting cells by mitogens to pass from $G_0$ to $G_1$ (Kelly *et al.*, 1983; Campisi *et al.*, 1984). In complementary experiments, c-*myc* RNA levels fall dramatically when cells withdraw from the cell cycle into $G_0$ or undergo terminal differentiation (for review see Spencer & Groudine, 1990). Steady state levels of c-*myc* RNA are subject to distinct control mechanisms: exceptionally short half-life of c-*myc* RNA (Dani *et al.*, 1984; Piechaczyk *et al.*, 1987), impaired maturation of the primary transcript (Eick, 1990), and a block to RNA-elongation at the first exon-intron border (Bentley & Groudine, 1986*a*; Eick & Bornkamm, 1986) as rapid means of c-*myc* regulation, and modulation of the rate of initiation as a late acting mechanism (Siebenlist *et al.*, 1988).

The structure of the gene with two major, $P_1$ and $P_2$ (Battey *et al.*, 1983), and two minor, $P_3$ and $P_0$ (Bentley & Groudine, 1986*a*; ar-Rushdi *et al.*, 1983; Hayday *et al.*, 1984; Bentley & Groudine, 1986*b*), sites of transcription initiation has been well characterised. Additionally, positive and negative regulatory elements have been found within or flanking the human and mouse c-*myc* gene (Yang *et al.*, 1986; Chung *et al.*, 1986; Remmers *et al.*, 1986; Lipp *et al.*, 1987; Kakkis & Calame, 1987; Hay *et al.*, 1987; Iguchi-Ariga *et al.*, 1988; Weisinger *et al.*, 1988; Asselin *et al.*, 1989; Hall, 1990) which may modulate initiation (Thalmeier *et al.*, 1989; Pietenpol *et al.*, 1990; Hall, 1990; Sacca & Cochran, 1990) and elongation of transcription (Bentley & Groudine, 1988; Miller *et al.*, 1989). Several transcription factors have been described which bind to sequences upstream of c-*myc* including NF1 (Siebenlist *et al.*, 1984), AP2 (Imagawa *et al.*, 1987), AP1, and octamer binding factors (Takimoto *et al.*, 1989; Hay *et al.*, 1989), NFkB (Duyao *et al.*, 1990), and a mouse plasmacytoma specific repressor protein (Kakkis *et al.*, 1989).

Stimuli from steroid hormones are generally considered to have a key-role in regulating cell proliferation and tissue development. Despite the increasing molecular data on steroid hormone-receptor complex action on individual response elements (for review see Beato, 1989), until now, the intricate cell-biological processes leading from hormonal signals to the modulation of cell proliferation remain poorly defined. Cell cycle arrest associated with c-*myc* down-regulation by steroid hormones has hitherto been described in lymphocytes and promyelocytes. Glucocorticoids block lymphocytes and lymphoma cells at the G1 phase of the cell cycle. Among a panel of known growth-related genes, only c-*myc* expression was reduced by dexamethasone (Yuh & Thompson, 1989). In a T lymphoblastic leukaemic cell line, immediate post-transcriptional down-regulation of c-*myc* has been demonstrated in response to glucocorticoids (Maroder *et al.*, 1990).

In the promyelocytic cell line HL60, 1,25-Dihydroxy-vitamin $D_3$ (1,25-$(OH)_2D_3$)-induced differentation along the monocyte lineage is preceded by a decrease in the steady state level of c-*myc* RNA (Reitsma *et al.*, 1983). The 1,25-$(OH)_2D_3$ effect on c-*myc* RNA was shown to occur at the transcriptional level (Simpson *et al.*, 1987).

Androgen analogues containing a 17α-methyl-testosterone backbone inhibit the proliferation (Sonnenschein *et al.*, 1989) and suppress the transformed phenotype (Wolf *et al.*, 1991) in the androgen responsive (Horoszewicz *et al.*, 1983; Schulz *et al.*, 1985; Berns *et al.*, 1986) human prostate carcinoma cell line LNCaP (Horoszewicz *et al.*, 1980). The androgen receptor of LNCaP cells carries a point mutation in the steroid binding domain but activates transcription in an androgen-dependent manner (Veldscholte *et al.*, 1990). LNCaP cells are considered to be the best-suited *in vitro* model of prostate cancer available (Thompson, 1990). Recently we could show that the synthetic androgen mibolerone represses anchorage-independent growth and concomitantly reduces the level of c-*myc* RNA in LNCaP cells (Wolf *et al.*, 1991). Here we have studied the details of hormonal c-*myc* repression. We demonstrate late transcriptional repression of the $P_1$, $P_2$ and $P_0$ promoters of c-*myc*.

### Materials and methods

### Cell culture and hormones

The prostate carcinoma cell line LNCaP (Horoszewicz *et al.*, 1980) was from the Human Tumor Cell Laboratory, Sloan Kettering Institute for Cancer Research, Rye, NY. LNCaP cells between passages 75 and 90 were used for the experiments described. Cells were maintained in RPMI medium as monolayers in the presence of 10% FCS and phenol red. For the preparation of seed stocks, cells were

Correspondence: D.A. Wolf.
[3]Present address: Ludwig Institute for Cancer Research, Box 595, S-75124 Uppsala, Sweden.
Received 2 September 1991; and in revised form 18 November 1991.

grown to 50 to 75% confluency before use. Hormones were added 48 h after seeding as ethanol solutions to give a final concentration as indicated in the figures. Synthetic androgen: 7α-17α-Dimethyl-19-nortestosterone (mibolerone; Upjohn). Antiandrogen: 6-chloro-6-dehydro-17α-acetoxy-1α,2α-methylene-progesterone (cyproterone acetate, CA; Schering).

*RNA extraction and Northern blot analysis*

Standard protocols were followed as described elsewhere (Wolf *et al.*, 1991).

*S1 mapping*

Single-stranded uniformly labelled DNA probes were prepared by primer extension of M13 clones, double-stranded probes by end-labelling with T4-polynucleotide kinase. Hybridisation of labelled DNA fragments to total RNA was carried out using a modification of the method of Berk and Sharp (1977). Hybridisation mixtures of 20 μl containing $10^5$ c.p.m. of the labelled probe (specific activity $10^8$ c.p.m. $\mu g^{-1}$), 40 μg RNA, 90% formamide, 400 mM NaCl, 40 mM Pipes, pH 6.5, 1 mM EDTA were denatured at 90°C for 5 min and immediately transferred to 58°C. After 15 h the hybridisation was terminated by addition of 180 μl ice-cold buffer containing 250 mM NaCl, 30 mM Na-acetate, pH 4.5, 2 mM Zn-acetate, 5% glycerol, and 400 units of nuclease S1 (Boehringer, Mannheim). The samples were incubated at 25°C for 1 h, extracted twice with phenol-chloroform-isoamylalcohol (25:24:1, v v v), and precipitated with ethanol. Protected DNA fragments were separated on 5% polyacrylamide gels containing 7 M urea.

*Nuclear run-on analysis*

Preparation of nuclear extracts and the hybridisation procedure were performed as described (Eick & Bornkamm, 1986) with slight modifications. $2 \times 10^8$ cells were scraped from culture dishes and washed twice in PBS. Cells were resuspended in 10 mM Tris-HCl, pH 7.4, 10 mM NaCl, 3 mM $MgCl_2$, 0.5% (v v) NP40 and incubated on ice for 5 min. The nuclear pellets were spun down at 500 *g* and washed by resuspension in 10 ml of the same buffer. The pelleted nuclei were resuspended in storage buffer (50 mM Tris-HCl, pH 8.3, 40% (v v) glycerol, 5 mM $MgCl_2$, 0.1 mM EDTA) and frozen in liquid nitrogen in portions of 100 μl corresponding to $2 \times 10^7$ nuclei. The nuclei were mixed with 100 μl reaction buffer (10 mM Tris-HCl, pH 8.0, 5 mM $MgCl_2$, 300 mM KCl, 0.5 mM of each ATP, CTP, GTP and 100 μCi of ($\alpha$-$^{32}P$) UTP (800 Ci mmol, Amersham)) and incubated for 20 min at 28°C. DNAseI was added to a final concentration of 10 $\mu g\ ml^{-1}$ and the incubation was continued for 5 min at 28°C. After addition of 200 μl STE buffer (100 mM Tris-HCl, pH 7.5, 50 mM EDTA, 0.5% SDS) and 20 μl proteinase K (10 $mg\ ml^{-1}$, preincubated at 37°C for 1 h) the samples were incubated for 1 h at 40°C. Nuclear transcripts were separated from unincorporated nucleotides on a Sephadex G-50 column equilibrated with 10 mM Tris-HCl, pH 7.5, 1 mM EDTA, 1% SDS. The labelled RNA was boiled for 10 min, chilled on ice and hybridised to DNA immobilised on nylon filters (PALL) in Church-buffer (0.5 M sodium phosphate, pH 7.1, 7% SDS, 0.1 mM EDTA) after preincubation of the filter in the same buffer. After hybridisation the filters were washed twice at 50°C in 0.1 × SSC, 1% SDS, twice in 2 × SSC containing 10 $\mu g\ ml^{-1}$ RNAase A at 25°C and finally once again in 0.1 × SSC, 1% SDS at 50°C. The filters were exposed to Kodak XAR-5 films using a Dupont Cronex Lightning Plus intensifying screen.

### Results

*Down-regulation of c-myc RNA in response to synthetic androgen is slow*

The effect of the synthetic androgen mibolerone (7α-17α-Dimethyl-19-nortestosterone) on c-*myc* expression in LNCaP cells was studied. Cells were incubated for 0-120 h in the presence or absence of $3.3 \times 10^{-9}$ M mibolerone followed by RNA extraction. Steady state c-*myc* RNA levels were analysed by Northern blotting using an exon 3-specific probe. The structure of the c-*myc* gene and all probes used in this work are depicted in Figure 1. When LNCaP cells are maintained in the presence of mibolerone a decline in c-*myc* RNA levels becomes clearly visible after 12–48 h (Figure 2a). After 72 h, the amount of c-*myc* RNA decreases to about 10% of control cells, but is still detectable in Northern blots. As shown in Figure 2b, the decrease of c-*myc* RNA is monophasic. In LNCaP control cells c-*myc* RNA levels begin to decline slightly after 24 h, and reach about 60% of the starting value after 120 h. RNA levels of the housekeeping enzyme glyceraldehyde-phosphate-dehydrogenase (GAPDH) did not change significantly during the experiment. Thus, the substantial reduction of c-*myc* RNA levels by androgen is a late effect. Androgen does not trigger any early down-regulation of c-*myc*. The effect of mibolerone is reversible: 48 h after withdrawal of the hormone c-*myc* steady-state RNA reached pretreatment levels (data not shown).

**Figure 1** Genomic map of the human c-*myc* gene depicting the probes used in this work. $P_0$, $P_1$, $P_2$, and $P_3$ designate the four c-*myc* promotors. Abbreviations of restriction enzymes: H = HindIII, Sm = SmaI, X = XhoI, P = PvuII, S = SacI, B = BstEII, C = ClaI, and E = EcoRI.

*Excess antiandrogen represses c-myc down-regulation*

If c-*myc* down-regulation is mediated by the androgen receptor, the effect of mibolerone must be suppressed by the simultaneous addition of antihormone which competes with androgen for specific receptor binding. In previous work (Wolf *et al.*, 1991) we found that a large excess of the antiandrogens cyproterone acetate (CA) or hydroxyflutamide is required to antagonise growth related effects of synthetic androgens in LNCaP cells. CA has a much lower affinity for the androgen receptor than synthetic androgens with a 17α-methyl-testosterone backbone (Wakeling *et al.*, 1981). The suppression of c-*myc* RNA levels is counteracted by the antiandrogen CA (Figure 2c) at the same concentration ratio (1:750 w w) at which the growth inhibiting effect of androgens on LNCaP cells is reversed (Wolf *et al.*, 1991). This finding indicates that the androgen receptor is involved in the signal transduction chain leading to c-*myc* down-regulation and to inhibition of proliferation by synthetic androgen.

*The levels of $P_1$, $P_2$, and $P_0$ transcripts decline, whereas $P_3$ transcripts remain almost constant*

In order to resolve the contribution of the four known c-*myc* promoters to the steady-state c-*myc* signal seen in Northern blots, we performed nuclease S1 protection assays with probes derived from the promoter region of the human c-*myc* gene. Southern analysis of the c-*myc* locus of LNCaP cells revealed no rearrangement or amplification (data not shown). Therefore, the expected sizes of S1 protected fragments were calculated on the basis of the germline configuration of the human c-*myc* gene. As shown in Figure 3a, the ratio of $P_2$:$P_1$:$P_0$ transcripts in control cells is about 80:20:3. This ratio is not changed in the presence of mibolerone. $P_1$, $P_2$ and

$P_0$ transcripts are reduced to the same extent (Figure 3a). In contrast, $P_3$ transcripts which comprise only 3–5% of the total c-*myc* transcripts in LNCaP cells, remain at a level almost equal to controls in the presence of mibolerone (Figure 3b). Regulation of the $P_3$-promoter independently of the $P_0$- $P_1$-, and $P_2$-promoter has recently been reported for the normal c-*myc* allele in the Burkitt's lymphoma cell line Raji (Eick *et al.*, 1990).

**Figure 2** Northern blot analysis of total RNA of LNCaP cells after treatment with the synthetic androgen mibolerone and the anti-androgen cyproterone acetate. LNCaP cells were cultivated in the presence (+) or absence (−) of $3.3 \times 10^{-9}$ M mibolerone (MIB) for various periods from 0–120 h. RNA was extracted and analysed on Northern blots (20 μg per lane) hybridised with a c-*myc* third exon specific probe labelled by random priming (ClaI-EcoRI, 1.4 kb, probe c in Figure 1). Subsequently, the probe was washed off and the filter was rehybridised with a glyceraldehyde-phosphate-dehydrogenase (GAPDH)-specific probe (Allen *et al.*, 1987) **a**. The autoradiograms were scanned densitometrically and c-*myc* RNA levels normalised to GAPDH RNA levels are shown schematically in a block diagram **b**. The effect of mibolerone is antagonised by cyproterone acetate (CA) ($1.8 \times 10^{-1}$ M). LNCaP cells were incubated in the presence of MIB and CA as indicated and RNA was extracted after 48 h **c**.

**Figure 3** C-*myc* promoter usage in LNCaP cells. The RNAs described in Figure 2a were subjected to S1 analysis with probes specific for the four c-*myc* promoters $P_0$, $P_1$, $P_2$, and $P_3$. RNA derived from the $P_0$-promoter was indirectly, RNA derived from promoter $P_1$ and $P_2$ directly visualised by a uniformly labelled, single-stranded SmaI-PvuII probe (probe a, Figure 1). $P_0$-RNA protected a fragment of 614 bases, $P_1$-RNA of 513 bases, and $P_2$-RNA of 351 bases **a**. Expression of $P_3$-RNA was studied in cells treated for 72 h with (+) or without (–) mibolerone. $P_3$-RNA was analysed with a double stranded XhoI-BstEII fragment (probe d, Figure 1) labelled at the BstEII site by T4 polynucleotide kinase. The probe protected a fragment of 322 bases corresponding to spliced $P_0$-, $P_1$-, and $P_2$-RNA **b**. The bands at 770 bases are specific for $P_3$-RNA. A long exposure of lanes 4 and 5 is shown at the right hand side. BL67 is a Burkitt's lymphoma cell line with a t(8;14) translocation and served as a positive control for expression of $P_3$-RNA (Eick *et al.*, 1985).

### *C-*myc *is down-regulated at the level of transcription initiation*

C-*myc* RNA is subject to an unusually rapid turnover (Dani *et al.*, 1984). In several cellular systems, c-*myc* RNA stability is the primary control mechanism to modulate steady-state levels (for review see Piechaczyk *et al.*, 1987). We have determined the contribution of posttranscriptional mechanisms to c-*myc* RNA repression in LNCaP cells and measured the stability of c-*myc* RNA after addition of actinomycin D. In the absence and presence (24 h) of mibolerone the half life of c-*myc* RNA was measured to be 15 to 20 min (data not shown). Therefore, a considerable contribution of a post-transcriptional mechanism is unlikely, and we have studied transcriptional control of c-*myc* RNA levels in nuclear run-on experiments. This method measures the density of actively transcribing RNA-polymerase II molecules on individual segments of the c-*myc* gene and allows to estimate the rate of c-*myc* RNA initiation and elongation.

In LNCaP control cells similar signals were obtained for the transcription rate of c-*myc* exon 1, 2, and 3 (Figure 4). However, the probe used for exon 1 has a size of 446 bases and is more than 3-fold smaller than the exon 2 and 3 probes with 1533 and 1405 bases, respectively. The relatively high transcription rate of c-*myc* exon 1 compared to exon 2 indicates that a fraction of RNA polymerases becomes blocked on the way to exon 2. A block to RNA-elongation in the c-*myc* gene has been described at the boundary of exon 1 to intron 1 in many different cellular systems (for review see Spencer & Groudine, 1990).

In the presence of mibolerone, the density and distribution of RNA polymerases on the c-*myc* gene remained unchanged for the first 3 h. Subsequently, transcriptional activity declined over a period of 2 days to about 10% of controls. The transcription of exon 1 and exon 2 slowed down with a similar rate. However the data presented in Figure 4 do not rule out a contribution of the RNA-elongation block for c-*myc* down-regulation between 6 and 12 h. In the presence of mibolerone and antihormone, transcription of c-*myc* was unaffected over a period of 2 days (data not shown).

The transcription rate for the PSA (prostate-specific antigen [Schulz *et al.*, 1988]) gene was increased 3 h after addition of mibolerone and subsequently declined towards pretreatment levels after 4 days (Figure 4). Thus, the decrease in c-*myc* transcription is not due to a general decrease in RNA synthesis concomitantly to inhibition of cell proliferation.

### Discussion

The synthetic androgen mibolerone is capable of triggering a set of fundamental changes in the growth behaviour of

**Figure 4** Transcriptional run-on activity of the c-*myc* gene in mibolerone treated LNCaP cells. 5 μg of single-stranded DNA fragments cloned in M13 and specific to c-*myc* exon 1 and 2 (fragments e and b, Figure 1) were separated in a 1.2% agarose gel and transferred to nylon filters by Southern blotting. The probes marked (+) detect c-*myc* sense transcripts and (−) detect antisense transcripts. The exon 3 probe is a double-stranded purified ClaI-EcoRI fragment (probe c, Figure 1). PSA is a probe for the prostate-specific antigen (1.5 kb EcoRI cDNA fragment [52]). The ethidium bromide stained gel before transfer is shown at the top. Filters were hybridised with nuclear run-on RNA ($10^7$ c.p.m./3 ml hybridisation buffer) from LNCaP cells treated without (0 h) or with mibolerone for 3–96 h.

LNCaP cells: inhibition of proliferation, abrogation of anchorage-independent growth, morphological change, and reduction of c-*myc* RNA levels (Wolf *et al.*, 1991). The signal transduction pathway from androgen binding to the ultimate cellular responses remains to be elucidated. In this report, the level of c-*myc* down-regulation by mibolerone in LNCaP cells has been analysed.

Mibolerone induces a late transcriptional repression of c-*myc* in LNCaP cells. The trancription rate for c-*myc* exon 1 and 2 decreased with a similar rate 3 to 6 h after addition of hormone. Thus, a reduced rate of RNA-elongation which has been described as fast control mechanism in c-*myc* down-regulation (Eick & Bornkamm, 1986) does not significantly contribute to c-*myc* repression in LNCaP cells. Mibolerone represses c-*myc* at the level of transcription initiation. The lag phase between hormone addition and the decline of c-*myc* transcription (>3 h) indicates that androgen receptor-mediated repression of c-*myc* involves additional regulatory steps.

Androgen regulation of c-*myc* has also been studied *in vivo*. C-*myc* RNA in the ventral prostate epithelial cells of rats increases nearly 4-fold within 1 day and 6- to 7-fold within 2 days after castration. The castration induces atrophy of prostatic epithelial cells while androgen treatment causes an increase in cell size and number. Administration of testosterone at the time of castration prevents the atrophy and the increases in c-*myc* RNA levels (Quarmby *et al.*, 1987). Similar observations were made studying the regression of androgen-dependent Shionogi mouse mammary carcinoma cells in castrated syngeneic animals. 3–6 days after castration the tumour mass began to regress accompanied by a continuous increase of c-*myc* RNA (Rennie *et al.*, 1988).

Repression of c-*myc* has also been reported for other steroid hormones. Glucocorticoids induce growth inhibition and c-*myc* repression in the human T lymphoblastic leukaemic cell line CCRF-CEM (Yuh & Thompson, 1989), in mouse lymphoma cells (Eastman-Reks & Vedeckis, 1986), in oestrogen-treated oviducts of immature chickens (Rories *et al.*, 1989), and in the murine lymphosarcoma cell line P1798 (Forsthoefel & Thompson, 1987). A direct transcriptional repression of the murine c-*myc* gene by binding of the glucocorticoid receptor complex to a response element upstream of exon 1 has been discussed as a possible mechanism of c-*myc* shutoff (Forsthoefel & Thompson, 1987).

The response elements for the glucocorticoid and androgen receptor share the imperfect palindrome GGTACANNNTGTTCT (Beato, 1989). In the androgen-regulated C3 gene of rat prostate androgen response is conferred by an element in the first intron (Claessens *et al.*, 1989). This element AGTACGTGATGTTCT differs in only two positions of the left hand part to the consensus sequence. The first intron of the c-*myc* gene harbours also a potential glucocorticoid/androgen receptor binding site GGTAGCAGCTGTTCT which diverges in two positions. All elements have the TGTTCT motif in common which has been shown to be functionally important for glucocorticoid and androgen response. Whether glucocorticoid and androgen receptors can bind to the described element in the first intron of c-*myc* has yet to be proven.

Alternatively, steroid-receptor complexes may exert their negative effect on c-*myc* expression without direct binding to a response element. The glucocorticoid receptor (GR) and the transcription factor AP1 can reciprocally repress one another's potency to activate transcription. In this particular antagonistic relationship, the negative factor (GR) does not displace the positive transcription factor (AP1) from its response element, but appears to interact directly with AP1 (Jonat *et al.*, 1990, Yang-Yen *et al.*, 1990; Schüle *et al.*, 1990). Thus, glucocorticoids may repress c-*myc* by direct binding to AP1 located at a binding site which has been described 330 bp upstream of the $P_1$-promoter. This region has been identified as negative element for c-*myc* transcription (Hay *et al.*, 1987; Takimoto *et al.*, 1989; Hay *et al.*, 1989). Whether the androgen receptor can reduce transcription initiation of c-*myc* via AP1 binding is not yet known.

*In vivo* androgen has a key-role in the maintenance of prostate cells. The hormone preserves the differentiated state of the cells and represses c-*myc*. High expression of c-*myc* accomplished by a retroviral vector results in benign hyperplasia in a reconstituted prostate model (Thompson *et al*., 1989). Thus, regulation of c-*myc* in prostate cells by androgen is evident. However, the precise role of c-*myc* in growth control of normal and neoplastic prostate cells remains to be elucidated.

We are grateful to B. Urlbauer for technical assistance and to W. Hörz, A. Polack and W. Hammerschmidt for critical reading of the manuscript. This work was supported by the Deutsche Forschungsgemeinschaft (SFB 190, Mechanismen und Faktoren der Genregulation).

### References

ALLEN, R.W., TRACH, K.A. & HOCH, J.A. (1987). Identification of the 37-kDa protein displaying a variable interaction with the erythroid cell membrane as glyceraldehyde-3-phosphate dehydrogenase. *J. Biol. Chem.* **262**, 649.

ASSELIN, C., NEPVEU, A. & MARCU, K.B. (1989). A cis-acting element in the promoter region of the murine c-*myc* gene is necessary for transcriptional block. *Oncogene*, **4**, 549.

AR-RUSHDI, A., NISHIKURA, K., ERIKSON, J., WATT, R., ROVERA, G. & CROCE, C.M. (1983). Differential expression of the translocated and the untranslocated c-*myc* oncogene in Burkitt lymphoma. *Science*, **222**, 390.

BATTEY, J., MOULDING, C., TAUB, R. & 5 others (1983). The human c-*myc* oncogene: structural consequences of translocation into the IgH locus in Burkitt lymphoma. *Cell*, **34**, 779.

BEATO, M. (1989). Gene regulation by steroid hormones. *Cell*, **56**, 335.

BENTLEY, D.L. & GROUDINE, M. (1986*a*). A block to elongation is largely responsible for decreased transcription of c-*myc* in differentiated HL60 cells. *Nature*, **321**, 702.

BENTLEY, D.L. & GROUDINE, M. (1986*b*). Novel promoter upstream of the human c-*myc* gene and regulation of c-*myc* expression in B-cell lymphomas. *Mol. Cell. Biol.*, **6**, 3481.

BENTLEY, D.L. & GROUDINE, M. (1988). Sequence requirements for premature termination of transcription in the human c-*myc* gene. *Cell*, **53**, 245.

BERK, A.J. & SHARP, P.A. (1977). Sizing and mapping of early adenovirus mRNAs by gel electrophoresis of S1 endonuclease-digested hybrids. *Cell*, **12**, 721.

BERNS, E.M.J.J., DE BOER, W. & MULDER, E. (1986). Androgen-dependent growth regulation of and release of specific protein(s) by the androgen receptor containing human prostate tumor cell line LNCaP. *Prostate*, **9**, 247.

CAMPISI, J., GRAY, H.E., PARDEE, A.B., DEAN, M. & SONENSHEIN, G.E. (1984). Cell-cycle control of c-*myc* but not c-*ras* expression is lost following chemical transformation. *Cell*, **36**, 241.

CHUNG, J., SINN, E., REED, R.R. & LEDER, P. (1986). Trans-acting elements modulate expression of the human c-*myc* gene in Burkitt lymphoma cells. *Proc. Natl Acad. Sci. USA*, **83**, 7918.

CLAESSENS, F., CELIS, L., PEETERS, B., HEYNS, W., VERHOEVEN, G. & ROMBAUTS, W. (1989). Functional characterization of an androgen response element in the first intron of the C3(1) gene of prostatic binding protein. *Biochem. Biophys. Res. Commun.*, **164**, 833.

COLE, M.D. (1986). The *myc* oncogene: its role in transformation and differentiation. *Annu. Rev. Genet.*, **20**, 361.

DANI, C., BLANCHARD, J.-M., PIECHACZYK, M., EL SABOUTY, S., MARTY, L. & JEANTEUR, P. Extreme instability of *myc* mRNA in normal transformed human cells. *Proc. Natl Acad. Sci. USA*, **81**, 7046.

DUYAO, M.P., BUCKLER, A.J. & SONENSHEIN, G.E. (1990). Interaction of an NF-kappa B-like factor with a site upstream of the c-*myc* promoter. *Proc. Natl Acad. Sci. USA*, **87**, 4727.

EASTMAN-REKS, S.B. & VEDECKIS, W.V. (1986). Glucocorticoid inhibition of c-*myc*, c-*myb*, and c-Ki-*ras* expression in a mouse lymphoma cell line. *Cancer Res.*, **46**, 2457.

EICK, D., PIECHACZYK, M., HENGLEIN, B. & 6 others (1985). Aberrant c-*myc* RNAs of Burkitt's lymphoma cells have longer half-lives. *EMBO J.*, **4**, 3717.

EICK, D. & BORNKAMM, G.W. (1986). Transcriptional arrest within the first exon is a fast mechanism in c-*myc* gene expression. *Nucleic Acids Res.*, **14**, 8331.

EICK, D. (1990). Elongation and maturation of c-*myc* RNA is inhibited by differentiation inducing agents in HL60 cells. *Nucleic Acids Res.*, **18**, 1199.

EICK, D., POLACK, A., KOFLER, E., LENOIR, G.M., RICKINSON, A.B. & BORNKAMM, G.W. (1990). Expression of P0- and P3-RNA from the normal and translocated c-*myc* allele on Burkitt's lymphoma cells. *Oncogene*, **5**, 1397.

FORSTHOEFEL, A.M. & THOMPSON, E.A. (1987). Glucocorticoid regulation of transcription of the c-*myc* cellular protooncogene in P-1798 cells. *Mol. Endocrinol.*, **1**, 899.

HALL, D.J. (1990). Regulation of c-*myc* transcription *in vitro*: dependence on the guanine-rich promoter element ME1a1. *Oncogene*, **5**, 47.

HAY, N., BISHOP, J.M. & LEVENS, D. (1987). Regulatory elements that modulate expression of human c-*myc*. *Genes & Develop.*, **1**, 659.

HAY, N., TAKIMOTO, M. & BISHOP, J.M. (1989). A FOS protein is present in a complex that binds a negative regulator of MYC. *Genes & Develop.*, **3**, 293.

HAYDAY, A.C., GILLIES, S.D., SAITO, H. & 4 others (1984). Activation of a translocated human c-*myc* gene by an enhancer in the immunoglobulin heavy-chain locus. *Nature*, **307**, 334.

HOROSZEWICZ, S.J., LEONG, S.S., MING CHU, T. & 8 others (1980). The LNCaP cell line a - a new model for studies on human prostatic carcinoma. *Prog. Clin. Biol. Res.*, **37**, 115.

HOROSZEWICZ, S.J., LEONG, S.S., KAWINSKI, E. & 5 others (1983). LNCaP model of human prostatic carcinoma. *Cancer Res.*, **43**, 1809.

IGUCHI-ARIGA, S.M.M., OKAZAKI, Y., ITANI, T., OGATA, M., SATO, Y. & ARIGA, H. (1988). An initiation site of DNA replication with transcriptional enhancer activity present upstream of the c-*myc* gene. *EMBO J.*, **7**, 3135.

IMAGAWA, M., CHIU, R. & KARIN, M. (1987). Transcription factor AP-2 mediates induction by two different signal transduction pathways: protein kinase C and cAMP. *Cell*, **51**, 251.

JONAT, C., RAHMSDORF, H.J., PARK, K.K. & 4 others (1990). Antitumor promotion and antiinflammation: down-modulation of AP-1 (Fos Jun) activity by glucocorticoid hormone. *Cell*, **62**, 1189.

KAKKIS, E. & CALAME, K. (1987). A plasmacytoma-specific factor binds the c-*myc* promoter region. *Proc. Natl Acad. Sci. USA*, **84**, 7031.

KAKKIS, E., RIGGS, K.J., GILLESPIE, W. & CALAME, K. (1989). A transcriptional repressor of c-*myc*. *Nature*, **339**, 718.

KELLY, K., COCHRAN, B.H., STILES, C.D. & LEDER, P. (1983). Cell-specific regulation of the c-*myc* gene by lymphocyte mitogens and platelet-derived growth factor. *Cell*, **35**, 603.

LIPP, M., SCHILLING, R., WIEST, S., LAUX, G. & BORNKAMM, G.W. (1987). Target sequences for cis-acting regulation within the dual promoter of the human c-*myc* gene. *Mol. Cell. Biol.*, **7**, 1393.

MARODER, M., MARTINOTTI, S., VACCA, A., SCREPANTI, I., PETRANGELI, E., FRATI, L. & GULINO, A. (1990). Post-transcriptional control of c-*myc* proto-oncogene expression by glucocorticoid hormones in human T lymphoblastic leukemic cells. *Nucleic Acids Res.*, **18**, 1153.

MILLER, H., ASSELIN, C., DUFORT, D. & 4 others (1989). A cis-acting element in the promoter region of the murine c-*myc* gene is necessary for transcriptional block. *Mol. Cell. Biol.*, **9**, 5340.

PIECHACZYK, M., BLANCHARD, J.-M. & JEANTEUR, P. (1987). C-*myc* regulation still holds its secrets. *Trends Genet.*, **3**, 47.

PIETENPOL, J.A., HOLT, J.T., STEIN, R.W. & MOSES, H.L. (1990). Transforming growth factor beta 1 suppression of c-*myc* gene transcription: role in inhibition of keratinocyte proliferation. *Proc. Natl Acad. Sci. USA*, **87**, 3758.

QUARMBY, V.E., BECKMAN, W.C., WILSON, E.M. & FRENCH, F.S. (1987). Androgen regulation of the c-*myc* proto-oncogene in rat prostate. *Mol. Endocrinol.*, **1**, 865.

REITSMA, P.H., ROTHBERG, P.G., ASTRIN, S.M. & 5 others (1983). Regulation of *myc* gene expression in HL-60 leukaemia cells by a vitamin D metabolite. *Nature*, **306**, 492.

REMMERS, E.F., YANG, J.-Q. & MARCU, K.B. (1986). A negative transcriptional control element located upstream of the murine c-*myc* gene [published erratum appears in *EMBO J.*, **5**, 3408 (1986)]. *EMBO J.*, **5**, 899.

RENNIE, P.S., BRUCHOVSKY, N., BUTTYAN, R., BENSON, M. & CHENG, T. (1988). Gene expression during the early phases of regression of the androgen-dependent Shionogi mouse mammary carcinoma. *Cancer Res.*, **48**, 6309.

RORIES, C., LAU, C.K., FINK, K. & SPELSBERG, T.C. (1989). Rapid inhibition of c-*myc* gene expression by a glucocorticoid in the avian oviduct. *Mol. Endocrinol.*, **3**, 991.

SACCA, R. & COCHRAN, B.H. (1990). Identification of a PDGF-responsive element in the murine c-*myc* gene. *Oncogene*, **5**, 1499.

SCHÜLE, R., RANGARAJAN, P., KLIEVER, S. & 5 others (1990). Functional antagonism between oncoprotein c-*Jun* and the glucocorticoid receptor. *Cell*, **62**, 1217.

SCHULZ, P., BAUER, H.W. & FITTLER, F. (1985). Steroid hormone regulation of prostatic acid phosphatase expression in cultured human prostatic carcinoma cells. *Hoppe-Seyler's Z. Physiol. Chem.*, **366**, 1033.

SCHULZ, P., STUCKA, R., FELDMANN, H., COMBRIATO, G., KLOBECK, H.-G. & FITTLER, F. (1988). Sequence of a cDNA clone encompassing the complete mature human prostate specific antigen (PSA) and an unspliced leader sequence. *Nucleic Acids Res.*, **16**, 6226.

SIEBENLIST, U., HENNINGHAUSEN, L., BATTEY, J. & LEDER, P. (1984). Chromatin structure and protein binding in the putative regulatory region of the c-*myc* gene in Burkitt lymphoma. *Cell*, **37**, 381.

SIEBENLIST, U., BRESSLER, P. & KELLY, K. (1988). Two distinct mechanisms of transcriptional control operate on c-*myc* during differentiation of HL60 cells. *Mol. Cell. Biol.*, **8**, 867.

SIMPSON, R.U., HSU, T., BEGLY, D.A., MITCHELL, B.S. & ALIZADEH, B.N. (1987). Transcriptional regulation of the c-*myc* protooncogene by 1,25-dihydroxyvitamin D3 in HL-60 promyelocytic leukemia cells. *J. Biol. Chem.*, **262**, 4104.

SONNENSCHEIN, C., OLEA, N., PASANEN, M.E. & SOTO, A.M. (1989). Negative controls of cell proliferation: human prostate cancer cells and androgens. *Cancer Res.*, **49**, 3474.

SPENCER, C.A. & GROUDINE, M. (1990). Control of c-*myc* regulation in normal and neoplastic cells. *Adv. Cancer Res.*, **56**, 1.

TAKIMOTO, M., QUINN, J.P., FARINA, A.R., STAUDT, L.M. & LEVENS, D. (1989). Fos jun and octamer-binding protein interact with a common site in a negative element of the human c-*myc* gene. *J. Biol. Chem.*, **264**, 8992.

THALMEIER, K., SYNOVZIK, H., MERTZ, R., WINNACKER, E.L. & LIPP, M. (1989). Nuclear factor E2F mediates basic transcription and trans-activation by E1a of the human MYC promoter. *Genes & Develop.*, **3**, 527.

THOMPSON, C.B., CHALLONER, P.B., NEIMAN, P.E. & GROUDINE, M. (1985). Levels of c-*myc* oncogene mRNA are invariant throughout the cell cycle. *Nature*, **314**, 363.

THOMPSON, T.C. (1990). Growth factors and oncogenes in prostate cancer. *Cancer Cells*, **2**, 345.

THOMPSON, T.C., SOUTHGATE, J., KITCHENER, G. & LAND, H. (1989). Multistage carcinogenesis induced by *ras* and *myc* oncogenes in a reconstituted organ. *Cell*, **56**, 917.

VELDSCHOLTE, J., RIS-STALPERS, C., KUIPER, G.G.J.M. & 7 others (1990). A mutation in the ligand binding domain of the androgen receptor of human LNCaP cells affects steroid binding characteristics and response to anti-androgens. *Biochem. Biophys. Res. Com.*, **173**, 534.

WAKELING, A.E., FURR, B.J.A., GLEN, A.T. & HUGHES, L.R. (1981). Receptor binding and biological activity of steroidal and non-steroidal antiandrogens. *J. Steroid. Biochem.*, **15**, 355.

WEISINGER, G., REMMERS, E.F., HEARING, P. & MARCU, K.B. (1988). Multiple negative elements upstream of the murine c-*myc* gene share nuclear factor binding sites with SV40 and polyoma enhancers. *Oncogene*, **3**, 635.

WOLF, D.A., SCHULZ, P. & FITTLER, F. (1991). Synthetic androgens suppress the transformed phenotype in the human prostate carcinoma cell line LNCaP. *Br. J. Cancer*, **64**, 47.

YANG, J.-Q., REMMERS, E.F. & MARCU, K.B. (1986). The first exon of the c-*myc* proto-oncogene contains a novel positive control element. *EMBO J.*, **5**, 3553.

YANG-YEN, H.F., CHAMBARD, J.C., SUN, Y.L. & 4 others (1990). Transcriptional interference between c-Jun and the glucocorticoid receptor: mutual inhibition of DNA binding due to direct protein-protein interaction. *Cell*, **62**, 1205.

YUH, Y.-S. & THOMPSON, E.B. (1989). Glucocorticoid effect on oncogene growth gene expression in human T lymphoblastic leukemic cell line CCRF-CEM. Specific c-*myc* mRNA suppression by dexamethasone. *J. Biol. Chem.*, **264**, 10904.